# Role of oxidative stress in calcific aortic valve disease and its therapeutic implications

**DOI:** 10.1093/cvr/cvab142

**Published:** 2021-04-21

**Authors:** Harry Z E Greenberg, Guoan Zhao, Ajay M Shah, Min Zhang

**Affiliations:** Department of Cardiology, Cardiovascular Division, King's College London British Heart Foundation Centre of Research Excellence, James Black Centre, 125 Coldharbour Lane, London SE5 9NU, UK; Department of Cardiology, The First Affiliated Hospital of Xinxiang Medical University, Heart Center of Xinxiang Medical University, Henan, China; Department of Cardiology, Cardiovascular Division, King's College London British Heart Foundation Centre of Research Excellence, James Black Centre, 125 Coldharbour Lane, London SE5 9NU, UK; Department of Cardiology, Cardiovascular Division, King's College London British Heart Foundation Centre of Research Excellence, James Black Centre, 125 Coldharbour Lane, London SE5 9NU, UK

**Keywords:** Aortic valve, Calcification, Reactive oxygen species, Oxidative stress, NADPH oxidases

## Abstract

Calcific aortic valve disease (CAVD) is the end result of active cellular processes that lead to the progressive fibrosis and calcification of aortic valve leaflets. In western populations, CAVD is a significant cause of cardiovascular morbidity and mortality, and in the absence of effective drugs, it will likely represent an increasing disease burden as populations age. As there are currently no pharmacological therapies available for preventing, treating, or slowing the development of CAVD, understanding the mechanisms underlying the initiation and progression of the disease is important for identifying novel therapeutic targets. Recent evidence has emerged of an important causative role for reactive oxygen species (ROS)-mediated oxidative stress in the pathophysiology of CAVD, inducing the differentiation of valve interstitial cells into myofibroblasts and then osteoblasts. In this review, we focus on the roles and sources of ROS driving CAVD and consider their potential as novel therapeutic targets for this debilitating condition.

## 1. Introduction

Calcific aortic valve disease (CAVD) is a progressive condition in which a normal tricuspid aortic valve or a congenitally abnormal bicuspid aortic valve becomes thickened, fibrosed, and calcified. The nature of this remodelling results in a spectrum of disease ranging from mild aortic sclerosis to severe aortic stenosis (AS). CAVD is currently the third most prevalent cardiovascular disease after coronary artery disease and hypertension.^1^ The prevalence of CAVD increases with age. Aortic valve sclerosis affects a quarter of over 65 years old,^1^ and progression from sclerosis to stenosis occurs in ∼2% of individuals per year.^2–4^ Most patients with established AS then develop moderate-to-severe symptoms requiring treatment.^1–3^ It is expected that in the absence of any preventative therapy, CAVD will represent an increasing disease burden as populations in developed countries age^5^ and as the prevalence of the associated cardiometabolic risk factors for CAVD namely obesity, type 2 diabetes, dyslipidaemia, and hypertension increase.^6^^,^^7^ Consequently, understanding the mechanisms underlying the initiation and progression of CAVD is vital for developing novel therapeutic targets.^8^

Amongst the various reported mediators of fibrocalcific valvular changes, there is emerging evidence of a critical, causative role for reactive oxygen species (ROS).^9–13^ This review will focus on the specific sources of ROS or ROS-mediated stress signalling driving CAVD, and examine their potential as novel therapeutic targets for this debilitating disease.

## 2. ROS in normal physiology

ROS are a group of highly reactive chemical forms of molecular oxygen, divided into free radical species (with at least one free electron) and non-radical (two electron) species.^14^ The most important examples of these are superoxide and hydrogen peroxide, respectively. Superoxide anions derive from the reduction of molecular oxygen in a reaction mediated by a variety of different enzymes at the cell membrane, cytoplasm, and in organelles, such as mitochondria, peroxisome, and endoplasmic reticulum.^14^^,^^15^ Subsequent spontaneous dismutation of superoxide or dismutation by superoxide dismutase (SOD) enzymes produces hydrogen peroxide. In turn, catalase converts hydrogen peroxide to water, although when partially reduced, hydrogen peroxide is converted to hydroxide ion and hydroxyl radical.

Highlights

- Calcific aortic valve disease (CAVD) is the third most common cardiovascular disease.
- There are currently no pharmacological therapies available for preventing, treating, or slowing the development of CAVD.
- Reactive oxygen species (ROS) have vital roles in the initiation and propagation of the disease.
- Targeting specific adverse sources of ROS including uncoupled nitric oxide synthases and reduced nicotinamide adenine dinucleotide phosphate oxidases 2 proteins represents a novel therapeutic avenue.

At physiological concentrations, ROS regulate cell growth, differentiation, senescence, migration, apoptosis, and autophagy.^16–18^ They also modulate a variety of metabolic processes including glycolysis, oxidative phosphorylation, and fatty acid synthesis.^15^^,^^19^ These responses are predominantly mediated via the oxidation of cysteine thiolate groups by hydrogen peroxide and of iron–sulphur clusters by superoxide on a wide-range of target proteins.^20^^,^^21^ These modifications regulate protein localization and function, as well as intermolecular and protein–protein interactions.^14^ Cysteine thiolate oxidation, in particular, leads to disulfide formation, cysteine persulfidation, and glutathionylation, which serve as important modulators of protein activity.^22^

Several mechanisms maintain ROS at physiological concentrations, including subcellular compartmentalization, SOD and catalase, peroxiredoxins, and the thioredoxin and glutathione systems which reverse the aforementioned cysteine residue modifications.^23^^,^^24^ The NRF2–KEAP1 system is also an important oxidant sensor in which oxidation of residues on KEAP1 leads to the up-regulation of NRF2 nuclear translocation where it acts as a transcription factor for a series of antioxidant proteins.^25^

## 3. ROS in cardiovascular diseases

Numerous cardiovascular disorders and diseases including endothelial dysfunction, hypertension, vascular calcification, atherosclerosis, cardiac remodelling, stroke, and diabetes are associated with an oxidative stress state in which ROS-producing enzyme activity and expression levels are up-regulated, whilst the expression and activity of antioxidant mechanisms are down-regulated.^26–28^ Within the cardiovascular system, three major sources of ROS are uncoupled nitric oxide synthases (NOS), reduced nicotinamide adenine dinucleotide phosphate oxidases (NOXs), and mitochondria.^29–31^ The roles of these sources of ROS in cardiovascular diseases, extensively reviewed elsewhere,^15^^,^^26–28^^,^^30^^,^^32^^,^^33^ are briefly described here.

### 3.1 Uncoupled NOS

NOS function as dimers which catalyse the transformation of L-arginine and molecular oxygen to nitric oxide (NO) and L-citrulline, requiring NADPH-derived electrons. NOS uncoupling occurs when there is a depletion of tetrahydrobiopterin (BH4), an obligatory co-factor that actions as an auxiliary electron donor in the above reaction.^34^^,^^35^ Uncoupling results in the production of superoxide as the enzyme switches from its classical NO synthase function to that of an NADPH-dependent oxidase.^36^ In turn, superoxide combines with NO to produce peroxynitrite, thereby reducing the bioavailability of NO.^11^^,^^34^^,^^37^^,^^38^ In the case of the endothelial NOS isoform (eNOS), in particular, this leads to impaired endothelial-derived NO-mediated relaxation of vascular smooth muscle with an associated increase in systemic vascular resistance and hypertension.^39^^,^^40^ Reduced NO bioavailability also results in detrimental vascular remodelling, impaired platelet aggregation, and leucocyte adhesion.^37^ Endothelial dysfunction secondary to BH4 deficiency is an early marker of, and a critical step in the development of atherosclerosis, as well as in diabetic micro- and macrovascular disease.^15^^,^^41–49^ Superoxide release from uncoupled eNOS and nNOS (neuronal NOS) has also been implicated in pressure-overload left ventricular hypertrophy and diastolic dysfunction.^42^^,^^50–53^

### 3.2 Nox proteins

NOXs generate ROS as their primary function, catalysing electron transfer from NADPH to O_2._^54–56^ There are seven mammalian NOX homologues (NOX1 to NOX5, Duox1, and Duox2) with NOX2 (also termed gp91^phox^) and NOX4 most widely expressed within the cardiovascular system.^31^^,^^55^ NOX2 is acutely activated by agonists such as angiotensin II, mechanical stimulation, and metabolic factors in a process that requires intracellular association between the transmembrane NOX2-p22^phox^ complex and the cytosolic subunits p47^phox^, p67^phox^, p40^phox^, and Rac1 to generate ROS.^54^^,^^57^ By contrast, NOX4 is constitutively active and regulated mainly by its own expression level,^58–61^ whilst it may also be activated by mechanical stretch and agonists such as TGF-β.^62^^,^^63^ Moreover, while NOX2 generates superoxide, NOX4 predominantly generates H_2_O_2_.^31^^,^^61^^,^^64^^,^^65^

Overwhelming evidence indicates that the pathophysiological roles of the NOXs are isoform and cell-type specific. NOX2 mediates the development of adverse cardiac fibrosis, cardiomyocyte hypertrophy, contractile dysfunction, and cardiomyocyte death induced by angiotensin II, pressure overload, or myocardial infarction.^30^^,^^31^^,^^59^^,^^66–75^ In the vasculature, NOX2 may also have important roles driving the initiation and progression of atherosclerosis.^15^^,^^32^^,^^76–81^

In contrast to the largely deleterious roles of NOX2, NOX4 may mediate protective signalling in the heart and vasculature.^60^^,^^82–87^ NOX4 protects against pressure overload-induced cardiac remodelling and dysfunction through paracrine preservation of myocardial capillary density,^60^^,^^88^ NRF2-dependent modulation of redox state,^89^ and enhancement of the integrated stress response.^90^ Cardiomyocyte NOX4 also maintains optimal mitochondrial function and cardiac performance during physiological exercise.^91^ Endothelial NOX4 protects against chronic pressure-overload induced cardiac remodelling,^84^^,^^86^ and AngII-stimulated myocardial fibrosis.^92^ In the vasculature, NOX4 protects against endothelial dysfunction, leucocyte adhesion, inflammation, and atherosclerosis.^82^^,^^87^ However, up-regulated vascular smooth muscle cell (VSMC) NOX4 correlates with VSMC dysfunction and plaque instability, whilst VSMC NOX4 deletion attenuates western-diet–induced atherosclerosis.^93^^,^^94^

### 3.3 Mitochondria

Mitochondria generate ROS (mitoROS) as natural by-products of oxidative phosphorylation and basal metabolic activity.^95^^,^^96^ MitoROS are tightly regulated by a number of mechanisms including the glutaredoxin, glutathione, and thioredoxin systems which support thiol redox homoeostasis, as well as by the anion carriers uncoupling protein 2 and 3 (UCP2 and 3) which, when activated by ROS, induce a proton leak that negatively influences ATP synthesis to diminish mitoROS production.^97^ In addition, mitochondrial SOD mediates dismutation of superoxide into hydrogen peroxide and subsequent decomposition into O_2_ and H_2_O occurs via the glutathione redox system.^98^ Additional regulation of mitoROS is mediated by the cytoplasmic GTPase dynamin-related protein-1 (DRP1), a key protein that also regulates mitochondrial fission.^99^

Several cardiovascular diseases are associated with dysfunctional mitochondrial activity, morphology, and localization, resulting in increased mitoROS production and impaired ROS-scavenging mechanisms. In cardiac tissue, mitoROS contribute significantly to post-ischaemic inflammatory infiltration, resulting in adverse cardiac hypertrophy, fibrosis and necrosis, and impaired excitation–contraction coupling.^100–104^ In atherosclerosis, mitoROS scavenging, genetic inhibition of mitoROS, or mito-targeted catalase each attenuate lesion progression and reduce inflammatory signalling and immune cell infiltration.^105–108^

## 4. ROS in the pathophysiology of CAVD

**Figure 1:**
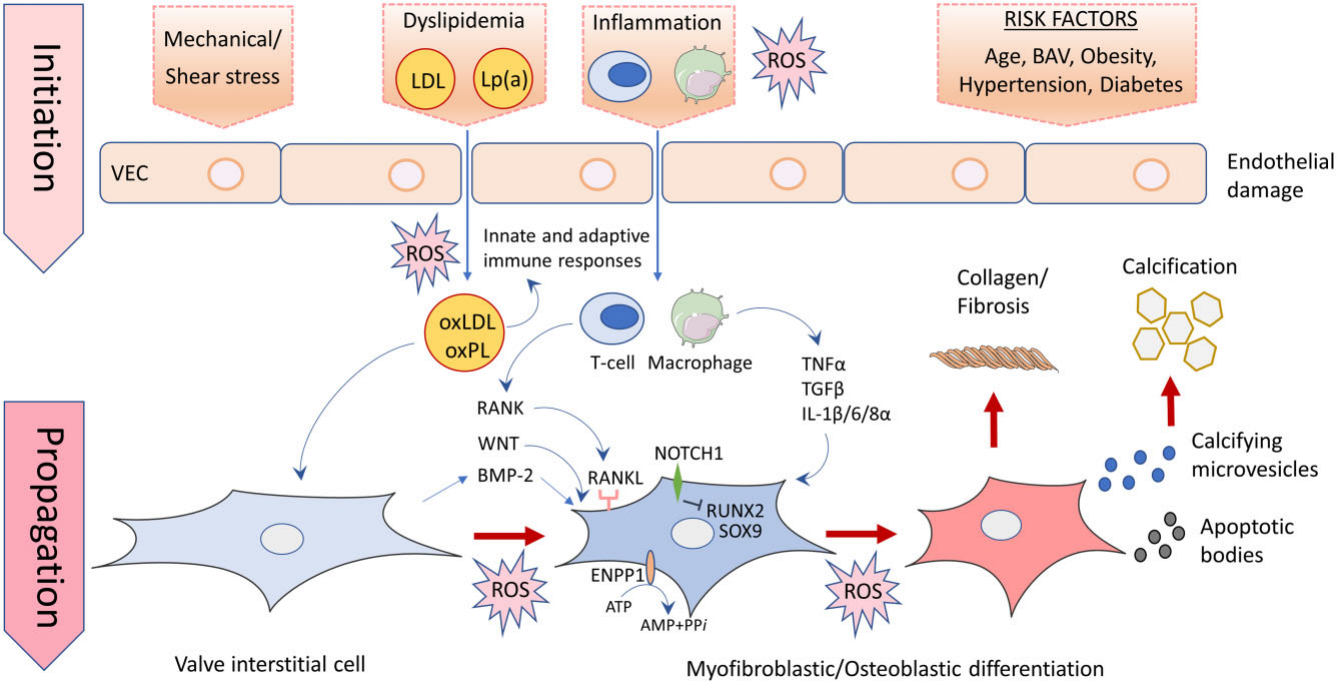
Pathogenesis of initiation and propagation of CAVD and potential roles of ROS. CAVD is initiated by valve endothelial cells (VEC) damage, triggered by diverse disease stimuli such as systemic inflammation, hyperlipidaemia, mechanical and shear stress, and other risk factors including ageing, hypertension, obesity, and diabetes. These permit the infiltration and oxidation of lipoproteins, and extravasation of inflammatory cells and subsequently innate and adaptive immune responses. In the propagation phase of the disease fibrosis and calcification occur, triggered by the release of cytokines that induce the transition of VICs into myofibroblasts and osteoblasts via the up-regulation of a number of fibrotic and osteogenic genes, respectively. Differentiation of VICs into myofibroblasts leads to leaflet thickening and fibrosis, due to extracellular matrix remodelling containing increased collagen and decreased elastin, forming a scaffold upon which amorphous deposition occurs. VIC transformation into osteoblasts is promoted further by a number of mechanisms including the down-regulation of and/or mutations in NOTCH1, which impair the ability of NOTCH1 to suppress osteogenic gene expression, up-regulated WNT/β-catenin signalling, and increased receptor activator of nuclear factor kappa B (RANK)/RANK ligand (RANKL) interactions. These differentiated VICs secrete calcifying microvesicles that promote calcium phosphate nucleation within valve leaflets. Apoptotic bodies released from VICs driven to programmed cell death by inflammatory cytokines and up-regulated ectonucleotidase expression act as a nidus for calcium and phosphorous crystal deposition. Adverse accumulation of ROS from a variety of sources mediates the oxidation of infiltrating lipids and inflammatory response in the initiation phase of CAVD. ROS are also critically implicated in the propagation phase of the disease, mediating the differentiation of VICs by up-regulating the expression of a series of osteogenic and fibrotic genes via a number of intracellular signalling cascades outlined in the main text. ATP, adenosine triphosphate; AMP, adenosine monophosphate; BAV, bicuspid aortic valve; BMP2, bone morphogenic protein2; ENPP1, ectonucleotide pyrophosphatase/phosphodiesterase family member 1; IL, interleukin; LDL, low-density lipoprotein; Lp(a), lipoprotein a; ROS, reactive oxygen species; NOTCH1, notch homolog 1, translocation-associated; PPi, inorganic pyrophosphate; RUNX2, runt-related transcription factor 2; SOX9, SRY-box 9; TGF-β, transforming growth factor beta; TNFα, tumour necrosis factor alpha.

It is now widely-recognized that CAVD is the manifestation of an active disease process involving multiple cellular mechanisms rather than the output of a passive, degenerative process.^1^^,^^8^^,^^109–111^ Increasing evidence points to critical roles of ROS in the initiation and propagation phases of CAVD (*Table 1* and *Figure 1*). Understanding these roles requires a brief review of the cellular mechanisms driving CAVD, that are extensively reviewed elsewhere.^1^^,^^111–116^

**Table 1:** Summary of animal and human studies investigating ROS in CAVD

Experimental models/tissues	*In vitro*/*in vivo*/*ex vivo*	Observations	Sources of ROS	Inhibitors tested	ROS assay	Ref
Animal studies						
LDLr^−/−^/ApoB^100/100^ mice fed normal chow	*In vivo* and *in vitro*	Hypercholesterolaemia induces CAVD in a subset of mice. Stenotic valves in hypercholesterolaemic mice demonstrate increased superoxide levels.	–	–	DHE	^9^
LDLr^−/−^/ApoB^100/100^ mice fed a western-type diet	*In vivo* and *in vitro*	Western-type diet induces hypercholesterolaemia and aortic valve lipid infiltration and deposition, and apoptosis leading to CAVD.	NOX2	Pioglitazone	–	^223^
Chronic Ang II infusion mice fed a hypercholesterolaemic diet	*In vivo* and *in vitro*	Chronic infusion of Ang II and hypercholesterolaemia induce oxidative stress within AVs, resulting in leaflet thickening, and ECM remodelling.	–	MnBuOE	–	^205^
Cultured porcine aortic valve interstitial cells	*In vitro*	TGF-β1 induces ROS production and calcium nodule formation via Smad and MAPK pathways.	–	–	DCF	^203^
Cultured porcine aortic valve interstitial cells	*In vitro*	TGF-β1 induces superoxide production which partly mediates *in vitro* calcification. Co-application with NO donors scavenges superoxide, reducing calcification.	–	DETA-NONOate SNP peg-SOD	DHE	^247^
Cultured porcine aortic valve interstitial cells	*In vitro*	Application of osteogenic medium induces valvular fibrosis and calcification mediated by ROS, collagen deposition, and up-regulation of fibronectin, OPN and Runx2 β-catenin accumulation.	NOX2	Celastrol	DHE	^13^
Cultured porcine aortic VECs and porcine aortic valve tissue	*Ex vivo* and *in vivo*	TNFα increases VEC intracellular oxidative stress, as well as superoxide and H_2_O_2_ synthesis. TNFα or H_2_O_2_ decrease nitric oxide synthesis by reducing eNOS expression. This results in myofibroblastic activation, calcification and changes in ECM composition and structure.	Uncoupled NOS and NOX2 Mitochondria	L-NAME BH4 Apocynin peg-SOD	DCF DHE Fluoro H_2_O_2_ MitoSOX Red^TM^	^209^
Rabbits fed high cholesterol diet + vitamin D	*In vivo* and *in vitro*	Superoxide and H_2_O_2_ levels are increased around calcifying foci and potentiate the progression of AV calcification. AV calcification improves by reducing H_2_O_2_ levels with lipoic acid.	NOX2, NOX4	Lipoic acid Tempol	DHE DCF	^10^
Rabbits fed high cholesterol diet + vitamin D	*In vivo* and *in vitro*	High cholesterol + vitamin D induces celastrol-sensitive increases in ROS which drives the development of CAVD and maladaptive cardiac remodelling.	NOX2	Celastrol	DHE	^13^
Cultured bovine aortic VICs	*In vitro*	Application of LPS induces oxidative stress, ALP overexpression, VIC calcification and ECM remodelling.	XOS	Allopurinol L-arginine	–	^236^
Human studies						
AV tissue from patients undergoing valve replacement surgery for symptomatic AS	*In vitro*	Superoxide and H_2_O_2_ levels are increased near calcified regions. SOD activity, expression of all 3 SOD isoforms, and catalase are significantly decreased in peri-calcific regions.	Uncoupled NOS	L-NAME	DHE DCF Lucigenin- chemiluminescence	^11^
AV tissues from patients with stenosis or sclerosis collected at surgery or autopsy	*In vitro*	Increases in ROS production are noted around calcifying foci in human sclerotic or stenotic AV.	NOX2	peg-SOD peg-Catalase	DHE	^10^
AV tissue and isolated cultured human VICs from patients with CAVD	*In vitro*	CAVD tissue demonstrates nitrotyrosine accumulation and increased peroxynitrite levels. SOD and catalase expression and activity are down-regulated. H_2_O_2_ induces impaired DNA-damage responses, profibrotic and pro-osteogenic signalling leading to osteogenic differentiation and calcification in isolated VICs	–	Adenoviral delivery of SOD/Catalase	–	^12^
AVs and isolated VICs from patients undergoing heart transplantation, valve replacement, or from deceased donor hearts.	*In vitro*	In sclerotic and stenotic valves, nitrotyrosine is diffusely distributed with areas of higher intensity. Dityrosine is only observed in stenotic tissue. TGF-β induces α-SMA up-regulation and aortic valve remodelling.	–	MnBuOE	–	^205^
Human VICs obtained from a normal healthy donor and two donor patients with calcified aortic valves	*In vitro*	In isolated human VICs reduction in antioxidant enzymes results in H_2_O_2_-induced increases in RUNX2 and OPN mRNA expression.	–	CNPs	DCF	^204^
Calcified human aortic valves obtained from adults undergoing valve replacement surgery	*Ex vivo*	Superoxide accumulates in calcified regions of the valves and in the fibrosa endothelium. Fibrosa-specific loss of SOD1 expression confers the fibrosa as the preferential site of superoxide accumulation.	–	–	DHE	^209^
Isolated aortic valve endothelial cells isolated from bicuspid valves explanted from patients with AS undergoing valve replacement	*In vitro*	Reduced expression of the antioxidants GPX3 and SRXN1 are observed in ECs isolated from BAVs leading to increased oxidative stress susceptibility.	–	–	–	^206^
Human aortic VICs isolated from patients undergoing AVR	*In vitro*	VICs incubated with Lp(a) develop increased ROS formation and undergo significant calcium deposition.	Mitochondria	–	DHE mitoSOX^TM^ Red	^225^
Primary and cultured human VICs isolated from stenotic AVs from patients undergoing valve replacement	*In vitro*	DRP1 overexpression, indicative of mitochondrial dysfunction, is observed, mediating OGM-induced VIC calcification.	Mitochondria	–	–	^228^
Myocardial biopsies from patients with AS undergoing elective valve replacement	*Ex vivo*	Myocardial biopsies demonstrate increased markers of the mitochondrial UPR, potentially indicating the presence of oxidative stress	Mitochondria	–	–	^226^
Isolated human VICs obtained from valves explanted from patients undergoing aortic valve replacement	*In vitro*	Application of Inorganic phosphate induces calcification of isolated human VICs.	SSAO	LJP1586	–	^234^

### 4.1 Initiation of CAVD

The progressive processes of leaflet fibrosis and calcification are initiated by damage to the endothelial cells lining the aortic valve.^117^^,^^118^ This damage, triggered by diverse risk factors including ageing, obesity and hypertension, systemic inflammation, and mechanical and shear stress, permits the infiltration, deposition, retention, and subsequent oxidation of lipoproteins, such as low-density lipoprotein (LDL) and lipoprotein(a) (Lp(a)).^119–123^ These events result in a chronic inflammatory response mediated by innate and adaptive immune responses,^118^^,^^124–127^ sustained and promoted by local neovascularization induced by vascular endothelial growth factor secreted from infiltrating mast cells.^128–132^ These vessels display augmented expression of vascular and intercellular adhesion molecules and endothelial selectin that further promote the inflammatory response.^133^^,^^134^

### 4.2 Propagation of CAVD

In the propagation phase of the disease fibrosis and calcification become the driving forces.^112^^,^^116^ Cytokines secreted from macrophages and T cells, such as TNFα, IL-1β, IL-6, IL-8α, insulin-like growth factor-1, and TGF-β,^1^^,^^112^^,^^135–139^ induce the transition of the predominant aortic valve cell type, namely valve interstitial cells (VICs), into myofibroblasts and osteoblasts.^1^^,^^140–146^ VICs are a heterogeneous population of fibroblast-like cells which are important physiological regulators of valve structure.^114^ Cytokines induce VIC differentiation by up-regulating the expression of several osteogenesis pathway genes including runt-related transcription factor 2 (Runx2), low-density lipoprotein receptor-related protein 5 (Lrp5), distal-less homeobox 5 (Dlx5), SRY-box 9 (SOX9), and muscle homeobox protein MSX2, as well as bone morphogenic protein 2 (BMP2)—a potent osteogenic differentiation factor, and the osteoblast marker proteins osteopontin (OPN), osteocalcin, and osteonectin.^137^^,^^144^^,^^145^^,^^147–157^ Direct activation of Toll-like receptors 2 and 4 by oxidized LDL also induces VIC BMP2 expression.^124^^,^^158^^,^^159^

Osteogenic VIC transformation is then promoted further by the down-regulation of and/or mutations in NOTCH1, which impair the ability of NOTCH1 to suppress Runx2 and SOX9 expression.^143^^,^^144^^,^^159^^,^^160^ Furthermore, up-regulated WNT/β-catenin signalling, reductions in anti-osteogenic microRNAs, such as miRNA-30b, and increased receptor activator of nuclear factor kappa B (RANK)/RANK ligand interactions all serve to promote the differentiation of VICs into osteoblast-like cells by up-regulating the expression of osteoblast-related genes.^112^^,^^143^^,^^144^^,^^146^^,^^152^^,^^156^^,^^159^^,^^161–168^

Differentiated VICs then secrete microvesicles containing ectonucleotidases, promoting calcium phosphate nucleation within valve leaflets.^1^^,^^169^^,^^170^ VIC overexpression of ectonucleotidases, such as Ectonucleotide pyrophosphatase 1 (ENPP1), alkaline phosphatase (ALP) and 5′-nucleotidase also drive valvular mineralization by generating inorganic phosphate and adenosine,^171–173^ and in the case of ALP, by hydrolyzing inorganic pyrophosphate and de-phosphorylating OPN, natural inhibitors of calcium phosphate deposition.^113^^,^^148^

Apoptotic bodies released from VICs driven to programmed cell death by inflammatory cytokines, such as TGF-β1 and ENPP1-mediatiated depletion of the key-cell survival signal ATP, also act as a nidus for calcium and phosphorous crystal deposition.^138^^,^^154^^,^^171^^,^^174^ In a subset of patients, heterotopic ossification mediated by the recruitment of bone-marrow-derived circulating osteogenic progenitor cells and endothelial progenitor cells occurs resulting in the formation of lamellar bone within the aortic valve leaflets.^175–178^

Alongside this mineralization process, leaflet thickening and fibrosis occur. This is mediated by the differentiation of VICs into myofibroblasts that induce extracellular matrix (ECM) remodelling by secreting excess collagen and increasing their expression of matrix metalloproteinases.^179^ These changes form a vital scaffold upon which hydroxyapatite nucleation and progressive amorphous deposition occur.^109^^,^^113^^,^^156^^,^^176^^,^^180–184^ Fibrotic changes are driven, in particular, by RANK/RANKL stimulation and by angiotensin II which is produced by chymase released from mast cells and by ACE delivered via LDL infiltration.^162^^,^^185–189^ The resulting extracellular matrix has relatively decreased elastin content and excessive disorganized collagen content which significantly alters leaflet biomechanics, leading to a higher mechanical load that in itself directly encourages further myofibroblastic differentiation in a positive feedback loop.^113^^,^^114^^,^^190^

### 4.3 Systemic inflammation and oxidative stress in CAVD

Analysis of plasma from patients with known AS reveals evidence of a systemic, pro-inflammatory state within which oxidative stress occurs. Accordingly, reduced levels of C4 indicating active complement pathway activation are observed alongside increased circulating pyroglutamic acid, an intermediate only generated when levels of the antioxidant glutathione are depleted.^191^

In a recent cohort study using thiobarbituric acid to assess for systemic lipid peroxidation and 2,4-dinitrophenylhydrazine to assess for the oxidative modification of plasma proteins, elevated oxidative stress was observed to correlate with the severity of AS, as reflected by mean aortic valve area and mean and maximum aortic gradients.^192^ Levels of oxidative stress also correlate with impaired systemic fibrinolysis, again suggesting that the oxidative stress in CAVD patients might not simply reflect a localized phenomenon.^192^ Evidence from animal models of CAVD also supports a putative role for systemic inflammation in CAVD. In both wild-type mice and in the atherosclerotic ApoE*3Leiden mouse model, intraperitoneal LPS induces AV thickening, but not calcification, suggesting a role for systemic inflammation in the early stages of CAVD.^193–195^

There are conflicting data on the association between serum levels of C-reactive protein (CRP), a marker of systemic inflammation, and CAVD. Several relatively small cohort studies (*n* ≤ 141) have identified a correlation between CRP levels and the presence and, in some studies, the severity of CAVD.^196–199^ However, a significantly larger cohort study (*n* = 5621) performed over a 5-year period, found that C-reactive protein is in fact not associated with baseline incidence of AS, progression to aortic sclerosis, or progression to AS.^200^

### 4.4 Localized valvular ROS signalling in CAVD

The majority of the evidence demonstrating roles for oxidative stress in CAVD derives from studies using aortic valve tissue and isolated VICs that together indicate a localized inflammatory response within which ROS signalling occurs. Indeed, a study that used computed tomography–positron emission tomography on patients with varying degrees of CAVD, observed localized valvular uptake of the tracers ^18^F-sodium fluoride and ^18^F-fluorodeoxyglucose that assessed for valvular calcification and inflammation, respectively.^201^ The degree of local valvular tracer uptake was observed to be correlated with the severity of disease.^201^

Pre-clinical and human studies reportedly demonstrate increased local ROS levels in CAVD. Many of these use fluorescent dyes to indicate ROS accumulation which possess varying degrees of specificity. Thus, whilst dihydroethidium (DHE) is a relatively specific indicator of superoxide, 2′7′-dichlorofluorescein diacetate (DCFH-DA) reacts with hydrogen peroxide and itself induces superoxide production, dismutation of which leads to self-amplification of DCF fluorescence.^202^ Caution is, therefore, required when interpreting results using this assay. The specificity of the commonly used chemiluminescence-based techniques, such as lucigenin-enhanced chemiluminescence for superoxide, have also been questioned, although when low concentrations are used, concerns regarding redox cycling in which lucigenin can react with oxygen to produce superoxide, are mitigated.^202^ The variety of assays used by the studies reviewed in this article are summarized in *Table 1*.

Around 30% of hypercholesterolaemic LDLr^−/−^ApoB^100/100^ mice fed a normal diet develop AS, with elevated aortic valve superoxide present both prior to CAVD developing, and more abundant in mice that subsequently develop AS.^9^ In isolated cultured porcine VICs, TGF-β1 induces ROS production subsequently leading to calcium nodule formation in a signalling cascade involving P38 MAPK and MEK1/2/ERK1/2 pathways.^203^ These findings are replicated by exogenous ROS application, which promotes VIC calcification by up-regulating fibrotic and osteogenic gene expression, indicating that oxidative stress may precede the differentiation of VICs to an osteoblastic phenotype. In rabbits fed a high cholesterol diet, both superoxide and H_2_O_2_ levels are increased in and around calcifying aortic valve, with AV calcification reversed by reducing H_2_O_2_ levels with lipoic acid.^10^

Importantly, ROS have also been implicated in human CAVD. In explanted valves from patients with established AS, superoxide and H_2_O_2_ levels are markedly increased near the calcified regions of the valve.^11^ This accumulation is in part due to a marked reduction in the peri-calcific activity and expression of all three SOD isoforms as well as catalase.^11^^,^^12^ Along similar lines, reduction in antioxidant enzymes results in hydrogen peroxide-induced increases in Runx2 and OPN mRNA expression in isolated human VICs.^204^

Increased ROS levels are also seen in valves from patients with aortic sclerosis. Here, peroxynitrite and nitrogen dioxide-generated nitrotyrosine levels are increased alongside superoxide and hydrogen peroxide.^12^ Together, these induce DNA damage, trigger dysfunctional DNA-repair mechanisms, and promote early VIC phenotypic alteration via up-regulation of AKT signalling leading to Runx2 and MSX2 overexpression as well as *in vitro* calcification. These changes are reversed with adenoviral delivery of superoxide dismutase and catalase.^12^ In explanted AV valves from patients with CAVD, nitrotyrosine is distributed throughout the sclerotic leaflets, with localized areas of high intensity also noted.^205^ In contrast, dityrosine is only observed in stenotic valves indicating more advanced oxidation occurring as disease progresses.^205^

Finally, in bicuspid aortic valves (BAV) from patients with AS undergoing surgical replacement, application of hydrogen peroxide triggers DNA damage and apoptosis in isolated valvular endothelial cells (ECs).^206^ Interestingly, increased levels of DNA damage and sustained apoptosis signalling are observed in bicuspid compared with tricuspid valves, secondary to molecular differences in oxidative stress susceptibility with reduced expression of the antioxidants glutathione peroxidase 3 and sulfiredoxin noted in BAV ECs.^206^

### 4.5 NOS-derived ROS in CAVD

Since uncoupled NOS primarily generate superoxide, it is feasible that the pro-inflammatory milieu in the initiation of CAVD may drive NOS uncoupling and thereby contribute to increases in superoxide levels observed in CAVD.^10^^,^^11^^,^^29^^,^^207^ Indeed, proteomic analyses of calcified aortic valves reveal decreased expression of HSP90, part of a complex with endothelial NOS, the dissociation of which can cause uncoupling of eNOS, leading to the production of ROS and endothelial dysfunction.^208^

In support of this hypothesis, the arginine analogue L-NAME, a NOS inhibitor, reduces superoxide production by over 50% in calcified human aortic valves.^11^ Data from animal models likewise support a role for uncoupled NOS-derived ROS in CAVD. In cultured porcine aortic valve ECs and in porcine aortic valve leaflets, exogenous TNFα and H_2_O_2_ induce eNOS uncoupling leading to increases in superoxide and H_2_O_2_ levels.^209^ Application of TNFα and H_2_O_2_ also reduces eNOS expression resulting in a reduction in NO synthesis as measured using the Griess assay. This in turn drives disorganization of the extracellular matrix and valvular calcification.^209^ These changes are partially reversed in the presence of the NOS inhibitor L-NAME, NOX inhibitor apocynin, or PEG-SOD and are fully reversed by the eNOS co-factor BH4, confirming NOS uncoupling as a key mediator in this model of CAVD.

**Figure 2:**
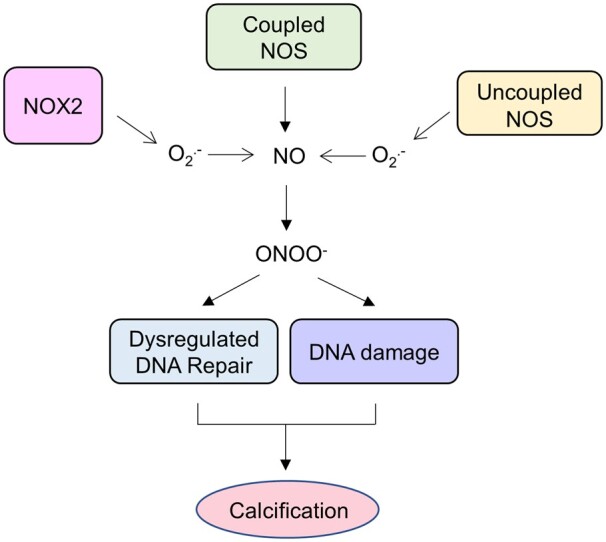
Nitric oxide synthase in the pathophysiology of calcific aortic valve disease. Uncoupling of endothelial nitric oxide synthase (eNOS) reduces nitric oxide bioavailability and increases superoxide $O_{2}^{-}$(O_2_) generation. In combination with superoxide generated from other sources, such as nicotinamide adenine dinucleotide phosphate oxidase 2 (NOX2), there is increased peroxynitrite (ONOO^−^) formation as it reacts with residual NO. In a positive feedback loop, this induces further NOS uncoupling via peroxynitrite-mediated oxidation of the obligatory nitric oxide synthase co-factor tetrahydrobiopterin. Peroxynitrite is also implicated in evoking DNA damage and dysregulated DNA repair responses leading to the up-regulated expression of osteogenic genes and valvular calcification.

Reduced NO bioavailability within diseased valve leaflets further increases the burden of ROS by decreasing NO-mediated quenching of superoxide. Indeed, alongside eNOS uncoupling, several other mechanisms are responsible for reducing NO synthesis in CAVD including mechanical stress-induced down-regulation of eNOS expression,^210^^,^^211^ and valvular endothelial-mesenchymal transition which occurs as a result of TGF-β1 stimulation,^212^ the constituent elements of the ECM,^213^ shear and mechanical stress,^214^ and protein S-glutathionylation—the consequence of an imbalance between reduced and oxidized glutathione.^215^ Hypercholesteraemia also decreases the expression and activity of eNOS,^216^^,^^217^ and therefore taken together these mechanisms reduce the availability of NO, resulting in increased superoxide levels driving valvular myofibroblast proliferation and extracellular matrix production.^216–220^ Moreover, enhanced levels of superoxide from other sources such as NOX2 (described in detail below) result in increased peroxynitrite formation as it reacts with residual NO. In a positive feedback loop, this induces further eNOS uncoupling via peroxynitrite-mediated oxidation of BH4.^10^^,^^207^^,^^221^^,^^222^ Peroxynitrite is also implicated in evoking DNA damage and dysregulated DNA repair responses within cultured human aortic VICs leading to the up-regulated expression of Runx2 and MSX2 and subsequent *in vitro* calcification^12^ (*Figure 2*).

### 4.6 NOX-derived ROS in CAVD

Recent evidence has emerged that NOX-derived ROS are critically involved in the development of CAVD. This is particularly so in animal models of CAVD, although conflicting evidence emerges with regards to the specific isoforms involved. In hypercholesteraemic mice fed a Western-type diet in order to induce CAVD, increases in NOX2 and p22^phox^ mRNA levels are observed within harvested valve leaflets, whilst no changes are observed for NOX4, eNOS, catalase, or SOD.^223^

Our recent studies with cultured primary porcine aortic VICs reveal that application of osteogenic medium (OGM) results in marked fibrosis and calcification associated with increased expression of NOX2, collagen, fibronectin, pro-osteogenic β-catenin accumulation, OPN, and Runx2.^13^ Additionally, *in vivo* evidence for NOX2-mediated CAVD is also shown in a rabbit model of CAVD fed cholesterol-enriched chow and vitamin D (HC + VitD). HC + VitD-treated rabbits develop thickened and fibrosed valve leaflets with increased calcium deposits.^10^^,^^13^ Phenotypically, this translates into significant decreases in AV area accompanied by enhanced transvalvular peak and mean jet velocity, ventricular dilatation, and contractile impairment as well as exaggerated cardiac hypertrophy.^13^ NOX2, p22^phox^, and its regulator protein disulfide isomerase are strongly expressed around calcification foci in HC+VitD rabbit aortic valves, coincident with the sites of highest hydrogen peroxide levels and the osteoblast differentiation marker OPN.^10^ Analyses of NOX1 and NOX4 mRNA expression also demonstrate increases in NOX4 expression in HC+VitD rabbits. Taken together therefore, these data support roles for NOX, especially NOX2-derived ROS in animal models of CAVD.

Nevertheless, in human CAVD, roles for NOX-derived ROS are less clear. In one study using stenotic valve tissue acquired from patients undergoing aortic valve replacement surgery or healthy tissue acquired post-mortem, NOX1 mRNA expression was below detectable limits, whilst expression of NOX2 and NOX4 mRNA did not differ significantly between normal valves and non-calcified regions of stenotic valves.^11^ Moreover, NOX2 and NOX4 mRNA levels in calcified regions of stenotic valves were significantly decreased compared with normal valve tissue. In addition, using a lucigenin-enhanced chemiluminescence assay to assess superoxide production, application of exogenous NADPH also produced similar increases in superoxide in tissue homogenates derived from calcified and non-calcified valves.^11^ Importantly, recent data has revealed that NADPH–dependent lucigenin chemiluminescence is an imperfect method to examine NOX activity.^224^

In contrast to these findings, a subsequent study revealed markedly increased NOX2 and p22^phox^ subunit expression around calcifying foci in human sclerotic and, to a greater degree, in human stenotic AV leaflets collected <6 hours post-mortem and surgery, respectively.^10^ Indeed, these findings have been confirmed in our recent study that clearly demonstrates NOX2 up-regulation in human CAVD.^13^ In normal valve tissue, immunohistology reveals relatively low expression of NOX2 whereas diseased valves exhibit intense NOX2 accumulation around the calcified regions. Western blotting demonstrates increased levels of NOX2 protein compared with non-calcified valves, accompanied by higher expression of Runx2.^13^

### 4.7 Mitochondrial ROS in CAVD

Recent evidence indicates a potential role for mitoROS in the development of CAVD. In isolated cultured human VICs, application of Lp(a) generates superoxide production from mitochondria as indicated by an increase in mitoSOX™ Red fluorescence, with Lp(a) application subsequently inducing *in vitro* VIC calcification.^225^ Notably MitoSOX^™^ Red fluorescence transiently increases after 1 h incubation with Lp(a), and then normalizes at 4 h. Thus, the significance of this observation is unclear given the temporary nature of the finding, although mitoROS might be important in the initiation of VIC calcification. Moreover, whether inhibiting mitoROS abrogates Lp(a)-induced VIC calcium deposition was not examined.

In isolated porcine aortic VICs, application of TNFα induces acute, peg-SOD-sensitive increases in mitochondrial ROS indicated by an increase in MitoSOX Red fluorescence, with the peak effect observed at 30 min.^209^ Moreover, in *ex vivo* porcine aortic valve leaflets, 21-day treatment with TNFα likewise induces peg-SOD-sensitive increases in mitoROS production in VECs located in the ventricular endothelium.^209^ Importantly, this study did not produce any direct experimental evidence implicating mitoROS as a propagator of CAVD and thus the observed increases in mitoROS may simply represent associations.

Myocardial biopsies from patients with AS demonstrate increased markers of the mitochondrial unfolded protein response, indicating a dysfunctional mitochondrial protein-folding environment. This environment is likely the result of cellular stress conditions, including up-regulated ROS and oxidative stress that results from and contributes to mitochondrial dysfunction in CAVD.^226^ Myocardial biopsies from patients with AS also demonstrate reduced expression of fatty acid translocase—a key enzyme involved in fatty acid oxidation, and the increased expression of glucose transporters 1 and 4. Decreases are also observed in the expression of the fatty acid binding proteins FABPpm and H-FABP, the β-oxidation protein medium chain acyl-coenzyme A dehydrogenase, the Krebs cycle protein α-ketoglutarate dehydrogenase, and the oxidative phosphorylation protein ATP synthase.^227^ Complex I of the electron transport chain is also down-regulated. Together these changes suggest a metabolic shift from fatty acid to glucose utilization, and, more generally, suggest further evidence of general mitochondrial dysfunction in patients with CAVD.^227^ However, the extent to which this metabolic shift results from and/or contributes to mitoROS in initiating CAVD remains unclear with no direct data linking the two. Moreover, it is important to point out that these data derive from myocardial tissue alone and thus whether similar abnormalities occur in the aortic valve tissue of these patients likewise requires further investigation.

In primary human VICs obtained from patients with established AS undergoing valve replacement surgery, DRP1 immunoreactivity is detected in calcified valve tissue. Similarly, in cultured human VICs treated with OGM containing inorganic phosphate and L-ascorbic acid for three weeks, DRP1 mRNA is significantly increased.^228^ DRP1 siRNA reduces human osteogenic medium-induced VIC calcification, indicating an important role for this mitochondrial regulator protein in regulating VIC calcification *in vitro*.^228^ These findings together intimate that mitochondrial dysfunction is an important feature of CAVD.

### 4.8 Other sources of ROS in CAVD

Several studies have identified semicarbazide-sensitive amine oxidase (SSAO), also known as vascular adhesion protein-1 as an additional deleterious source of ROS in CAVD. SSAO generates H_2_O_2_ from endogenous amines such as histamine and dopamine, with their activity and expression up-regulated in atherosclerosis, obesity, and diabetes.^229–231^

SSAO expression and activity are also increased in human CAVD where serum and valvular expression levels correlate with disease severity.^232^^,^^233^ Here, enzyme activity is up to seven times higher than it is in healthy parts of the same valves as assessed by H_2_O_2_ production following the application of the SSAO substrate benzylamine.^234^ In addition, a significant correlation of SSAO expression with oxidative stress is observed, where the enzyme is co-localized with calcified regions of aortic valve tissue. SSAO mRNA levels are positively correlated with mRNA levels of the NOX subunit p22^phox^ and the nuclear enzyme poly(ADP-ribose) polymerase which is activated following DNA damage and oxidative stress.^235^ In addition, inhibition of SSAO activity with LJP1586 attenuates calcification induced by high concentrations of phosphate in primary cultures of VICs isolated from aortic valves of CAVD patients.^234^

In bovine aortic VICs, application of LPS induces increased ALP expression and VIC calcification.^236^ This is associated with increased expression of xanthine oxidase (XOS), which, under oxidative stress, produces superoxide.^237^ Co-application of allopurinol which inhibits XOS, reverses LPS-induced ALP overexpression.^236^

## 5. Targeting ROS in CAVD

**Figure 3:**
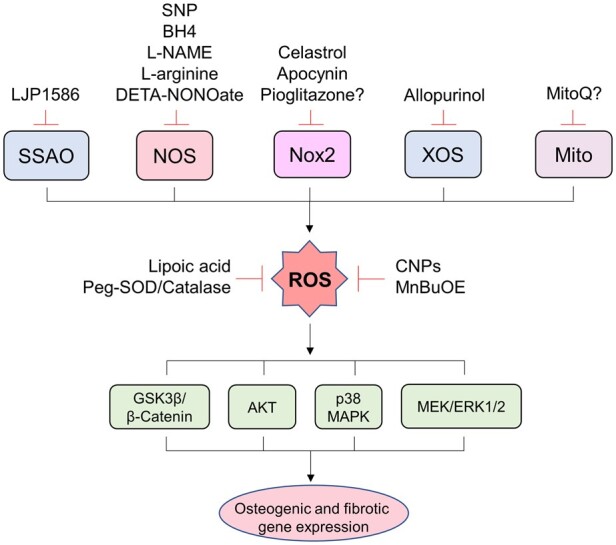
The adverse sources of ROS and potential therapeutic implications in CAVD. Pre-clinical and human studies implicate several adverse sources of ROS in the pathophysiology of CAVD. Intracellular ROS accumulation induces the activation of a variety of signalling cascades that increase the expression of osteogenic and fibrotic genes, phenotypically transforming aortic valvular interstitial cells and leading to valvular calcification and fibrosis. Several potential therapeutic strategies that seek to inhibit ROS-mediated oxidative stress have been suggested. AKT, protein kinase B; BH4, tetrahydrobiopterin; CNPs, cerium oxide nanoparticles; DETA-NONOate, 2,2'-(2-Hydroxy-2 nitrosohydrazinylidene)bis-ethanamine; ERK, extracellular signal-regulated kinases; GS3β, glycogen synthase kinase 3 beta; L-NAME, L-NG-Nitroarginine methyl ester; MAPK, mitogen-activated protein kinase; MEK, mitogen-activated protein kinase; Mito, mitochondria; MnBuOE, Mn(III) meso-tetrakis (N-n-butoxyethylpyridinium-2-yl) porphyrin; NOS, nitric oxide synthase; NOX2, reduced nicotinamide adenine dinucleotide phosphate oxidase 2; Peg-Catalase, polyethylene glycol-catalase; Peg-SOD, polyethylene glycol-superoxide dismutase; ROS, reactive oxygen species; SSAO, semicarbazide-sensitive amine oxidase; SNP, sodium nitroprusside; XOS, xanthine oxidase.

There is a major unmet clinical need for novel pharmacological treatments capable of preventing or slowing the progression of CAVD. Several avenues using existing therapies have failed to show benefit in large randomized controlled trials (RCTs) including treatment with statins, antihypertensives, and drugs targeting phosphate and calcium metabolism.^1^^,^^111^^,^^115^^,^^238–243^ This failure partially results from the fact that by the time patients present with CAVD, the multifactorial, and self-perpetuating cellular mechanisms driving the disease have already been set in motion.^113^^,^^117^^,^^244–246^ As described above, specific ROS sources and ROS-mediated signalling may represent novel therapeutic targets given their roles in the initiation and propagation of the cellular mechanisms driving CAVD. Proposed strategies for inhibiting ROS-induced oxidative stress either systemically or locally in CAVD are therefore outlined below and summarized in *Table 1* and *Figure 3*.

### 5.1 Impairing lipid oxidation

As described, lipid oxidation is a key trigger of inflammation in the initiation of CAVD.^248^ Oxidized LDLs are increased in valves removed from patients with AS, with an association between the level of oxidized LDL and the extent and pace of aortic valve fibrosis and calcification.^189^^,^^245^^,^^249–251^ As carriers of oxidized phospholipids (OxPL), Lp(a) also have an important role in the initiation of inflammation seen in CAVD. Approximately one-third of patients with AS have elevated plasma Lp(a), with large genetic and cohort studies revealing that the higher the level, the faster CAVD progresses, with marked increases in the risk of requiring valve replacement or death from the disease.^140^^,^^141^^,^^225^^,^^245^^,^^252^^,^^253^

*In vitro* studies have demonstrated that LDL and Lp(a) oxidation promote the expression of osteogenic differentiation genes but also ROS-mediated VIC calcification. Isolated human aortic VICs cells incubated with either LDL or Lp(a) demonstrate calcification, with those treated with Lp(a) displaying a higher burden of calcium deposition and ROS formation.^225^ Moreover, progression of CAVD is significantly impaired in transgenic Ldlr^−/−^ mice that express a single-chain variable fragment of E06, a natural antibody which binds to the phosphocholine headgroup of OxPL, and blocks the uptake of oxidized low-density lipoprotein by macrophages.^254^

These data, therefore, suggest that diminishing the extent of lipid/phospholipid oxidation by lowering plasma LDL and/or Lp(a) levels might represent a therapeutic avenue for treating CAVD. Indeed, in Ldlr^−/−^Apob^100/100^ mice, inactivation of the mttp gene significantly impairs hypercholesterolaemia-induced oxidative stress, thereby reducing aortic valve lipid deposition, osteogenic signalling, and valvular calcification.^255^

Nevertheless, despite promising associations between statin use and lower prevalence of CAVD in observational studies,^256–262^ RCT data demonstrate no benefit for statin use in CAVD.^240^^,^^241^^,^^244–246^^,^^263–266^ Randomization to statin use is not only of no benefit in halting the progression of established CAVD but also does not reduce the incidence of CAVD in patients with no established diagnosis at the start of the clinical trials.^266^ In addition, as yet there are no RCT data on specific Lp(a)-lowering therapy, although an antisense oligonucleotide that specifically lowers Lp(a) levels is currently under investigation.^267^

The synthetic compound probucol, purported to inhibit LDL oxidation, has also been proposed as a novel treatment for CAVD.^268^^,^^269^ Indeed, probucol has been clinically proven to arrest the progression of atherosclerosis in the vasculature and in coronary artery restenosis following angioplasty.^12^^,^^270–273^ However, it is now clear that probucol likely mediates most of its effects via the induction of HO-1, rather than through direct inhibition of lipid oxidation.^274^

### 5.2 Antioxidants

Broad ROS scavenging with natural and synthetic ‘antioxidant’ compounds has been attempted by several groups for attenuating atherosclerosis and vascular calcification. These studies, reviewed extensively elsewhere,^275^ have investigated a variety of compounds, such as diosgenin, vitamins A–E, quercetin, 10-DHGD, and curcumin, each of which have been shown *in vitro* to scavenge ROS and/or up-regulate endogenous antioxidant mechanisms, subsequently attenuating inflammation and vascular calcification.^275^

Moreover, several large observational studies have suggested an inverse relationship between dietary ‘antioxidant’ intake such as α-tocopherol, β-carotene, and vitamins C and E, with cardiovascular morbidity and mortality.^273^^,^^276^^,^^277^ However, meta-analyses of randomized control trial data on the effects of vitamins B, C, E, and S or β-carotene have found identical rates of cardiovascular morbidity and mortality in the placebo and antioxidant groups.^273^^,^^278^^,^^279^

A number of reasons for these disappointing findings have been proposed including the inadequate length of follow-up periods and the dosing of antioxidants tested. In addition, given that ROS also mediate important physiological processes,^273^ generalized scavenging of ROS is unlikely to prove beneficial if it interrupts cellular homeostasis and cardiovascular physiology such as endothelial-mediated control of vascular tone by superoxide and hydrogen peroxide as well as platelet aggregation, angiogenesis, and immune cell activity.^15^ It is, therefore, plausible to hypothesize that given the failure of these compounds in atherosclerosis and vascular calcification, that they are unlikely to prove beneficial for those with CAVD.

### 5.3 Enhancing SOD and catalase activity

Reductions in antioxidant enzyme levels are observed in calcified aortic valve leaflets.^11^^,^^12^ Adenoviral delivery of SOD or catalase significantly reduces ROS-induced human VIC DNA damage, osteoblastic differentiation, and valvular calcification.^12^ Similarly, application of the cell-permeable polyethylene glycol-SOD (peg-SOD) or peg-catalase significantly decrease superoxide and H_2_O_2_ levels respectively in human stenotic AVs and in rabbits on a high cholesterol and vitamin D diet.^10^ Peg-SOD also inhibits TGF-β1-induced calcium nodule formation by 70% in porcine aortic VICs.^247^ Intriguingly, in porcine aortic VICs, peg-SOD actually promotes OGM-induced AVIC calcification,^13^ suggesting that this approach may only work in certain models of CAVD, or at a certain range of dosage.

Cerium oxide nanoparticles (CNPs) act as ROS scavengers by switching between Ce^3+^ and Ce^4+^ oxidation states^238^^,^^280^ and by acting as catalase and SOD-mimetics.^204^^,^^280–282^ In calcified human VICs, rod- and sphere-shaped CNPs scavenge H_2_O_2_  *in vitro* and decrease VIC differentiation as assessed by osteoblast marker expression.^204^ Recently, the manganese porphyrin‐based compound and SOD mimic MnTnBuOE‐2‐PyP5+ (MnBuOE) has been shown to inhibit aortic valve remodelling in human VICs and mouse models of CAVD.^205^ MnBuOE prevents TGF‐β-induced α‐SMA up-regulation in human VICs, and in hypercholesterolaemic mice with a chronic infusion of Ang II, co-treatment with MnBuOE reduces AV thickening and ECM remodelling by reducing collagen deposition and improving fibre alignment.^205^

It is important to point out that clinical trials adopting this approach, i.e. increasing SOD and catalase expression and/or activity must carefully consider the pleiotropic roles for ROS in regulating a wide-range normal physiological processes.^14^^,^^15^^,^^18^ It is reasonable to assume that any intervention which overcorrects for oxidative stress will unlikely prove beneficial, particularly so when considering the balance between local adverse ROS signalling and systemic increases in ROS driving CAVD.

### 5.4 Increasing NO bioavailability

Several studies have attempted to reduce the overall burden of ROS within the aortic valve by increasing the bioavailability of NO. Increasing NO bioavailability not only reduces the burden of ROS but also regulates Notch1 signalling and its nuclear localization to reduce the expression of osteogenic markers in VICs.^283^^,^^284^

In isolated porcine VICs, application of TGF-β1 induces superoxide production which partly mediates valve calcification.^247^ However, co-application with the NO donors DETA-NONOate and sodium nitroprusside (SNP) scavenges superoxide, reducing *in vitro* calcification.^247^ DETA-NONOate also inhibits OGM-induced VIC differentiation and matrix calcification in isolated porcine VICs.^285^ Moreover, pre-treatment with L-arginine, the precursor for NO synthesis, significantly attenuates the osteogenic differentiation of bovine aortic VICs exposed to the endotoxin LPS.^236^ In the presence of L-arginine, LPS-induced ALP expression and subsequent matrix calcification are reduced, alongside reductions in LPS-induced TNF-alpha, IL-6, and IL-1β expression. Pre-treatment with L-arginine also reduces xanthine oxidase expression, and markers of ECM remodelling including ADAMTSL4, basigin, and COL3A1.^236^ Finally, in calcified *ex vivo* human aortic valves exposed to TNFα, co-treatment with BH4 mitigates eNOS uncoupling, increasing NO bioavailability which reduces superoxide levels, thereby attenuates the expression of osteogenic genes.^209^

The above findings support, at least in principle, the proposal that increasing NO bioavailability might represent a novel approach for treating and/or slowing the progression of CAVD. Nevertheless, there is currently no clinical trial evidence on the effectiveness of this approach. In addition, part of the pleiotropic nature of statins are their ability to increase NO bioavailability via the stabilization of eNOS mRNA,^216^^,^^286–289^ and in a rabbit model of CAVD, statins reduce AV calcification via this mechanism.^216^ Given the aforementioned failure of statin therapy in clinical trials, different approaches to increasing NO bioavailability require further investigation. These might include organic nitrate therapy, administration of L-arginine or BH4, or using the K_ATP_ channel opener nicorandil, which also possesses a nitrate moiety. Renin-angiotensin system inhibitors such as ACE inhibitors might also slow the progression of CAVD by increasing NO bioavailability via up-regulating eNOS expression, reducing bradykinin breakdown, and by supressing NOX-generated superoxide.^290–294^

### 5.5 Inhibiting NOX2

Specific inhibition of only the deleterious sources of ROS in CAVD represents a more nuanced approach than the broad ROS scavenging approaches described above. To this end, inhibitors of NOX2 signalling have been used in animal models of CAVD, but with varying results. In porcine aortic valves, apocynin, which blocks phosphorylation of the obligatory NOX2 cytosolic component p47^phox 58,295,296^ but may also have non-specific antioxidant activity, only partly reduces TNFα-induced increases in superoxide and hydrogen peroxide.^209^ This is in contrast to isolated mice aortic myofibroblasts where apocynin markedly impairs TNF- and IL-1β-induced NOX2 ROS generation, a finding that is recapitulated with antisense oligonucleotides targeted against NOX2.^137^

In hypercholesterolaemic LDLr^−/−^/apoB^100/100^ mice fed a western diet, pioglitazone reduces aortic valve osteogenic signalling, aortic valve calcification, and improves cusp mobility, possibly due to a reduction of inflammation and oxidative stress that is associated with down-regulated NOX2 expression.^223^ The specific contribution of NOX2 to this improvement is not however clear, given that multiple other inflammatory mediators including TNF and IL-6 are also down-regulated by pioglitazone therapy.^223^

Interestingly, in isolated porcine VICs, treatment with celastrol, a pentacyclic triterpene naturally extracted from the roots of *Tripterygium wilflordii*, and a potent NOX inhibitor with higher potency against NOX2,^297^ decreases NOX2 and Runx2 protein levels, VIC ROS levels, and reduces calcium deposition.^13^ Pro-osteogenic accumulation of β-catenin is also reversed with celastrol, impairing NOX2-mediated inactivation of GSK3β, which in turn enables β-catenin degradation. These findings are replicated when isolated aortic VICs are transfected with adenoviral vectors expressing a short hairpin sequence targeted against NOX2,^13^ an important observation given that NOX-independent effects of celastrol are also described in the cultured cell line PLB-985.^298^ Of note, celastrol may have other global beneficial effects, such as anti-obesity and anti-inflammation properties,^299^^,^^300^ which are comorbidities of CAVD likewise characterized by an increase in ROS production mediated, at least in part, by NOX2 activation.^17^

Moreover, in a rabbit model of CAVD fed with HC + VitD, dietary celastrol markedly alleviates the degree of AS and improves cardiac dilatation, contractility, and function.^13^ Celastrol treatment also improves rabbit AV fibro-calcification, as indicated by decreases in collagen deposition, the expression of fibronectin, OPN, and the number of calcium deposits.^13^

Recently, celastrol has also been shown to suppress Runx2 and OPN expression and reverse calcification in cultured porcine aortic VICs exposed to a medium containing high concentrations of calcium and phosphate.^301^ Here, celastrol reverses calcium-induced up-regulation of BMP2‐BMPRII-Smad1/5 and Wnt/β‐catenin signalling pathways, preventing their respective nuclear translocation and modulation of osteogenic gene expression. These findings are replicated *in vivo* by intraperitoneal injection of celastrol in mice fed adenine to induce CKD and intraperitoneal vitamin D to induce valvular calcification.^301^ However, NOX2 involvement in this model of CAVD remains to be investigated.^301^

Together, these data suggest that NOX2 represents a novel therapeutic target in the treatment of CAVD with further work required to delineate its role in human CAVD. Given the role played by NOX2 in neutrophil function,^302^ future work targeting NOX2 in CAVD will need to consider whether this compromises innate immune responses, although previous work demonstrates that this only occurs with substantial NOX2 inhibition,^303^ thus safe therapeutic targeting of cardiovascular NOX2 could be feasible.

## 6. Conclusions and perspectives

CAVD is the end result of multiple active cellular and molecular mechanisms converging on the phenotypic change of aortic VICs. Oxidative stress is an important driver of these processes and targeted inhibition of the deleterious sources of ROS, particularly NOX2 and uncoupled eNOS, represent important avenues in the search for novel therapies. This targeted approach theoretically avoids interfering with the wide variety of normal cellular processes dependent on ROS signalling and may therefore yield better results for preventing or slowing the progression of CAVD than approaches non-specifically inhibiting redox signalling trialled thus far. Notably, in cancer medicine, a number of drugs targeting excessive ROS signalling that broadly up-regulate antioxidant mechanisms have thus far proved unsuccessful with unexpected toxic side effects often observed in normal tissues.^304^

Presently there is a lack of *in vivo* and clinical data on human CAVD investigating roles and sources of adverse ROS signalling driving the disease. Specifically, there is a need for adequately powered cohort observational studies that might establish if there is a relationship between the extent of valvular oxidative stress, the pace of disease progression, and its relation to clinical severity. Novel imaging modalities that assess the *in vivo* burden of valvular oxidative stress using redox-sensitive probes may therefore represent important tools.^305^^,^^306^ In addition, larger scale *in vitro* studies examining roles and sources of ROS in human CAVD will also be necessary given the relatively small number of patients recruited to each of the studies described in this review. These *in vitro* studies may wish to make use of novel, and more-specific fluorescent ROS probes^307^ and ROS-targeting nanotechnologies,^308^^,^^309^ when exploring roles of ROS signalling in human CAVD.

Despite evidence of a role for mitoROS in a range of cardiovascular diseases,^15^^,^^27^^,^^105^ there remains no direct evidence of a pathophysiological role for these in CAVD. Future work might seek to identify whether mitoROS indeed play a role and whether targeting these ROS with recently developed site-specific mitochondrial ROS inhibitors, such as MitoQ effects the development and/or progression of the disease whilst maintaining the range of normal metabolic functions reliant on normal levels of mitoROS.^310^ Importantly, excessive ROS production by non-mitochondrial sources (such as uncoupled NOS enzymes or NOXs) can trigger mitochondrial dysfunction and further ROS production—sometimes termed ROS-induced ROS release.^311^^,^^312^ Therefore, further work is needed to explore the cross-talk between ROS at distinct subcellular compartment in aortic valve cells and its contribution to CAVD.

Finally, it would appear likely that any effective therapy preventing or slowing the progression of CAVD needs to be initiated early in the evolution of the disease, rather than at the stage where a patient presents with severe AS. Identifying and targeting pivotal molecular pathways especially ROS-mediated canonical redox signalling at an early stage will prove challenging, and may require the use of state-of-the-art technologies, such as network medicine analysis and multi-omics mapping that generate transcriptional and protein expression signatures for the different stages of CAVD.^313^ Indeed, the feasibility of this approach has recently been demonstrated for CAVD,^314^ potentially an important step in characterizing the large number of cellular and molecular changes occurring as the disease develops and progresses.

**Conflict of interest:** none declared.

## Funding

This study was supported by the British Heart Foundation (PG/17/39/33027 to M.Z.; CH/1999001/11735 to A.M.S.), the National Natural Science Foundation of China (81470506), and Key Research Project of the Heart Center of Xinxiang Medical University (2017360).
